# Machine learning for post-acute pancreatitis diabetes mellitus prediction and personalized treatment recommendations

**DOI:** 10.1038/s41598-023-31947-4

**Published:** 2023-03-24

**Authors:** Jun Zhang, Yingqi Lv, Jiaying Hou, Chi Zhang, Xuelu Yua, Yifan Wang, Ting Yang, Xianghui Su, Zheng Ye, Ling Li

**Affiliations:** 1 Department of Endocrinology, Zhongda Hospital, School of Medicine, Southeast University, Nanjing, 210009 Jiangsu China; 2 Institute of Glucose and Lipid Metabolism, Southeast University, Nanjing, China; 3 Department of Endocrinology, Changji Branch, First Affiliated Hospital of Xinjiang Medical University, Xinjiang, 831100 China; 4 Department of Endocrinology, Hunan Provincial People’s Hospital, First Affiliated Hospital of Hunan Normal University, Changsha, 410005 Hunan China; 5 Department of Endocrinology, Yixing Second People’s Hospital, Wuxi, 214200 China; 6 Department of Endocrinology, Zhongda Hospital, School of Medicine, Institute of Pancreas, Southeast University, Nanjing, 210009 China

**Keywords:** Diseases, Endocrinology, Gastroenterology, Risk factors, Signs and symptoms

## Abstract

Post-acute pancreatitis diabetes mellitus (PPDM-A) is the main component of pancreatic exocrine diabetes mellitus. Timely diagnosis of PPDM-A improves patient outcomes and the mitigation of burdens and costs. We aimed to determine risk factors prospectively and predictors of PPDM-A in China, focusing on giving personalized treatment recommendations. Here, we identify and evaluate the best set of predictors of PPDM-A prospectively using retrospective data from 820 patients with acute pancreatitis at four centers by machine learning approaches. We used the L1 regularized logistic regression model to diagnose early PPDM-A via nine clinical variables identified as the best predictors. The model performed well, obtaining the best AUC = 0.819 and F1 = 0.357 in the test set. We interpreted and personalized the model through nomograms and Shapley values. Our model can accurately predict the occurrence of PPDM-A based on just nine clinical pieces of information and allows for early intervention in potential PPDM-A patients through personalized analysis. Future retrospective and prospective studies with multicentre, large sample populations are needed to assess the actual clinical value of the model.

## Introduction

Acute pancreatitis (AP) is one of the most common gastrointestinal diseases characterized by acute pancreas inflammation and acinar cell destruction. The global incidence of AP is increasing dramatically worldwide^1–3^. AP was originally thought to be a self-limiting disease. Most patients recover completely, but only about 20% may develop severe acute pancreatitis (SAP), with a mortality rate of around 30%^4,5^. However, diabetes, as a sequela of AP, has drawn attention in recent years. National Population-Based cohort studies reveal that the risk of PPDM-A is twofold higher than those without AP, and the occurrence of PPDM-A is observed across the spectrum of severity in AP^6–8^. Its prevalence has tripled in the past decade and is expected to reach 13.6 per 100,000 by 2050^9^. PPDM-A has gained more and more attention in the field of diabetes.

Pancreas contains both exocrine and endocrine parts. The exocrine pancreatic disease can lead to diabetes of the exocrine pancreas (DEP), the second most common type of new-onset diabetes in adults (surpassing type 1 diabetes)^10^. Furthermore, AP is considered the most common cause of DEP, and about 80% of pancreatitis-related DEP is due to AP^11^. This type of diabetes is characterized by impaired endocrine and exocrine functions, including significant glycemic drift, frequent episodes of hypoglycemia (fragile diabetes)^10^, and impaired digestion and absorption of nutrients^12,13^. It seriously threatens human health and places a heavy burden on health care^14,15^.

However, PPDM-A has not drawn sufficient attention and has often been misdiagnosed as T2DM^12,16^. Diabetes mellitus is highly heterogeneous and requires fine diagnostic staging for precise treatment. Therefore, screening high-risk patients is essential for developing PPDM-A prevention guidelines, delaying islet function damage, avoiding adverse outcomes, and improving the prognosis of PPDM-A.

In this study, we used a machine learning model to screen the most important clinical features for predicting PPDM-A and used these clinical features to construct a logistic regression model with L1 regularisation, obtaining good AUC and F1 values. In addition, we interpreted the predictions of the model using nomograms and Shapley values and provided personalized early prevention protocols. This study provides a valuable guide to the occurrence and prevention of PPDM-A.

## Results

### Study cohort and baseline characteristics

Between 1 October 2016 and 31 October 2021, 3477 admissions for AP were screened in hospital information system (HIS). After using the exclusion criteria described (Supplementary Fig. 1), 820 patients with AP without known diabetes were included in our study. Of these, two-thirds (n = 574) were randomly assigned to the training set, with the remaining one-third (n = 246) assigned to a validation cohort. Table 1 shows the baseline characteristics of the patients. The median age was 50 (38, 63) years. The proportion of males was 61.3% (n = 503). Biliary was the most common cause of AP. 484 (59%) patients presented with mild AP, 280 (34.1%) with moderate AP, and 56 (6.8%) with severe AP; 68 (8.3%) patients had PPDM-A, and they were more likely to be obese (20.7% vs. 9.2%, P = 0.005), presenting with hyperlipidemia and tending to have combined non-alcoholic fatty liver disease (NAFLD) (75% vs. 45.2%, P < 0.001). Smoking rates were higher in patients with PPDM-A than in those without DM.

**Table 1:** Patient demographics and clinical characteristics.

Characteristics	Total (n = 820)	PPDM-A (n = 68, 8.3%)	AP without DM (n = 752, 91.7%)	P value
Age, years	50 (38, 63)	49.5 (37, 56.8)	50 (38, 63)	0.480
Gender, male, n (%)	503 (61.3)	47 (69.1)	456 (60.6)	0.169
Length of stay, d	9 (7,14)	11 (7, 15)	9 (7, 14)	0.206
Obesity, n (%)	81 (9.9)	12 (20.7)	69 (9.2)	0.005
Family history of diabetes, n (%)	57 (7)	9 (13.2)	48 (6.4)	0.060
Smoking, n (%)	206 (25.1)	25 (36.8)	181 (24.1)	0.021
Drinking, n (%)	196 (23.9)	16 (23.5)	180 (23.9)	0.940
NAFLD, n (%)	391 (47.7)	51 (75)	340 (45.2)	< 0.001
SBP (mmHg)	130 (120, 144)	132 (120, 146.5)	130 (120, 144)	0.357
DBP (mmHg)	80 (72,88)	80 (76,87)	80 (72,88)	0.511
Infection (n, %)	361 (44)	34 (50)	327 (43.5)	0.300
Hypertension, n (%)	219 (26.8)	24 (35.3)	195 (26)	0.097
Severity, n (%)				
Mild	484 (59)	36 (52.9)	448 (59.6)	0.026
Moderate	280 (34.1)	22 (32.4)	258 (34.3)	
Severe	56 (6.8)	10 (14.7)	46 (6.1)	
Amylase (U/L)	560 (198.3, 1300)	269 (107.5, 985.3)	597.5 (216.5, 1300)	0.009
Admission glucose (mmol/L)	6.66 (5.6, 8.3)	8.56 (6.95–11.64)	6.52 (5.54, 8)	< 0.001
Ca (mmol/L)	2.20 (2.08, 2.31)	2.19 (2.04, 2.27)	2.20 (2.09, 2.32)	0.162
ALT (U/L)	51.1 (23, 148.9)	33.5 (21.1, 71.75)	53.45 (23, 155.9)	0.047
AST (U/L)	33 (19.6, 89.45)	27.1 (19.9, 54.03)	34.8 (19.6, 94.58)	0.139
ALP (U/L)	88.15(65.78, 134.2)	87 (62.65, 115.23)	88.85 (66, 135)	0.259
LDH (IU/L)	244.45 (188, 343)	282.5 (201.69, 384.75)	242 (190.7, 340)	0.235
CK (IU/L)	68 (45, 105)	69.5 (51.75, 111.98)	68 (44.08, 104.25)	0.231
BUN (mmol/L)	4.29 (3.3, 5.6)	4.7 (3.9, 6.3)	4.2 (3.2, 5.5)	0.013
Cr (umol/L)	65 (52, 77)	67.9 (56.25, 84)	64.4 (52, 76)	0.052
UA (umol/L)	295 (226, 363.9)	332.5 (276, 405.2)	289.85 (224, 357)	0.003
TG (mmol/L)	1.24 (0.83, 2.95)	3.53 (1.3, 8.77)	1.19 (0.8, 2.64)	< 0.001
TC (mmol/L)	4.41 (3.65, 5.54)	5.33 (4.19, 7.47)	4.35 (3.6, 5.37)	< 0.001
HDL-C (mmol/L)	1.02 (0.75, 1.3)	0.995 (0.41, 1.38)	1.02 (0.75, 1.29)	0.869
LDL-C(mmol/L)	2.40 (1.88, 3.06)	2.55 (1.70, 3.32)	2.40 (1.88, 3.05)	0.051
ANC, n (%)	36 (4.4)	6 (8.8)	30 (4)	0.120
APFC, n (%)	290 (35.4)	24 (35.3)	266 (35.4)	0.990

Values are given as median (IQR) or frequencies (percentages).

*SBP* systolic blood pressure, *DBP* diastolic blood pressure, *ALT* alanine transaminase, *AST* aspartate aminotransferase, *ALP* alkaline phosphatase, *LDH* lactate dehydrogenase, *NAFLD* non-alcoholic fatty liver disease, *Cr* creatinine, *BUN* blood urea nitrogen, *UA* uric acid, *TG* triglyceride, *TC* total cholesterol, *HDL-C* high-density lipoprotein cholesterol, *LDL-C* low-density lipoprotein cholesterol, *APFC* acute peripancreatic fluid collection, *ANC* acute necrotic collection.

### Feature extraction

**Figure 1:**
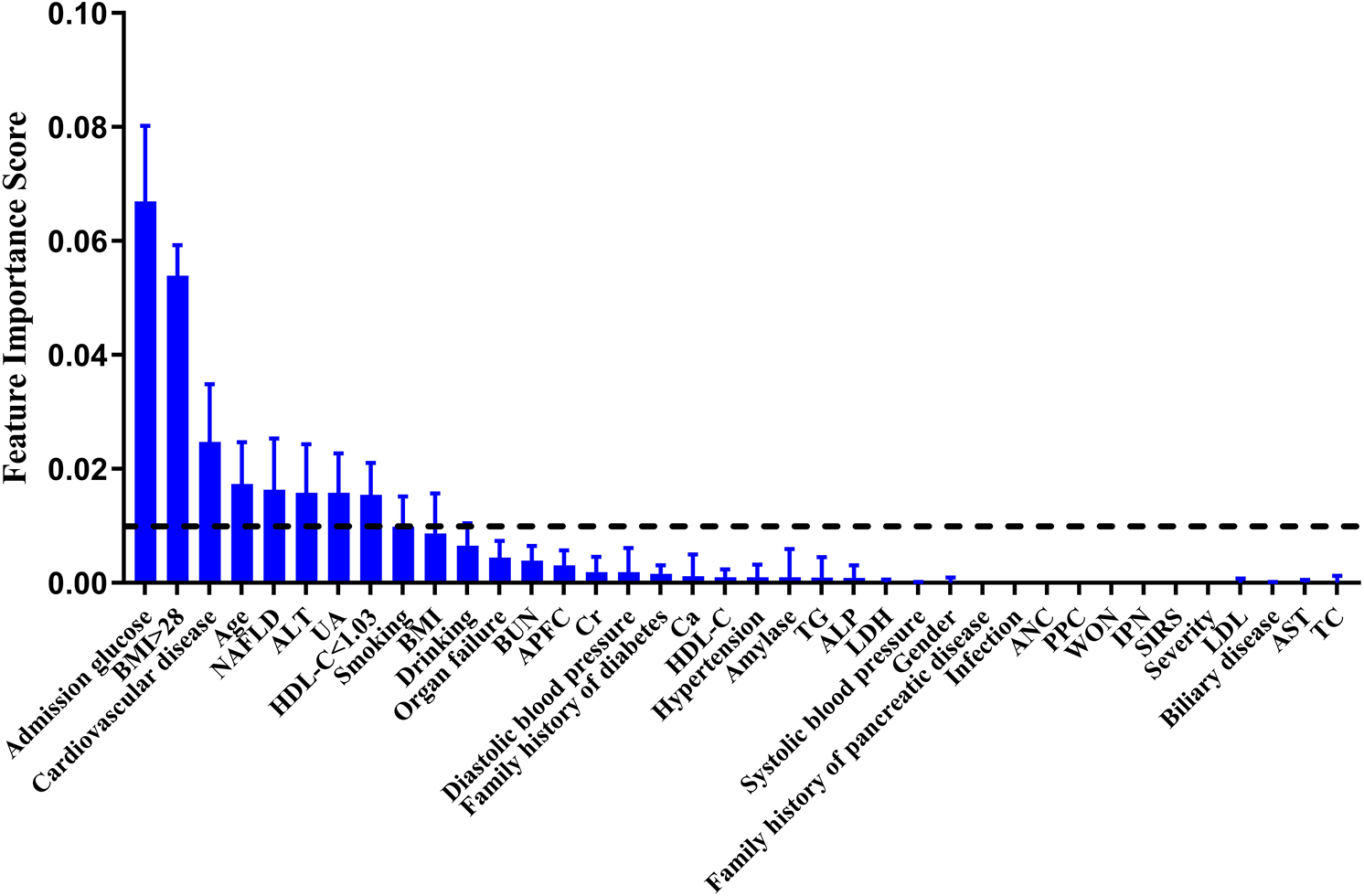
Core influencing factor screening. The mean values of the weights of the features were ranked by 1000 lasso regressions. Of these, nine features with Feature Importance Score > 0.01 were selected as core genes considered to be associated with PPDM-A.

Lasso regression (L1 regularized logistic regression) can be used for feature extraction in classification models. We performed 1000 randomly perturbed lasso regressions to extract the weights of 38 clinical features. By ranking the mean weights of these 38 features and using a threshold of 0.01, we obtained the nine most influential indicators on the classification of the model (Fig. 1), in order of Admission glucose, obesity (BMI > 28 kg/m^2^), cardiovascular disease (CVD), Age, NAFLD, alanine transaminase (ALT), uric acid (UA), HDL-C < 1.03 mmol/l, Smoking. In addition, indicators with a residual range above 0 included several features, drinking, organ failure, acute peripancreatic fluid collection (APFC), blood urea nitrogen (BUN), creatinine, hypertension, amylase, Ca. The results show that the two most influential factors in PPDM-A are still Admission glucose and obesity. These two indicators are also those associated with type 2 diabetes. It suggests that type 2 diabetes and PPDM-A share common risk factors.

### Algorithm performance

**Figure 2:**
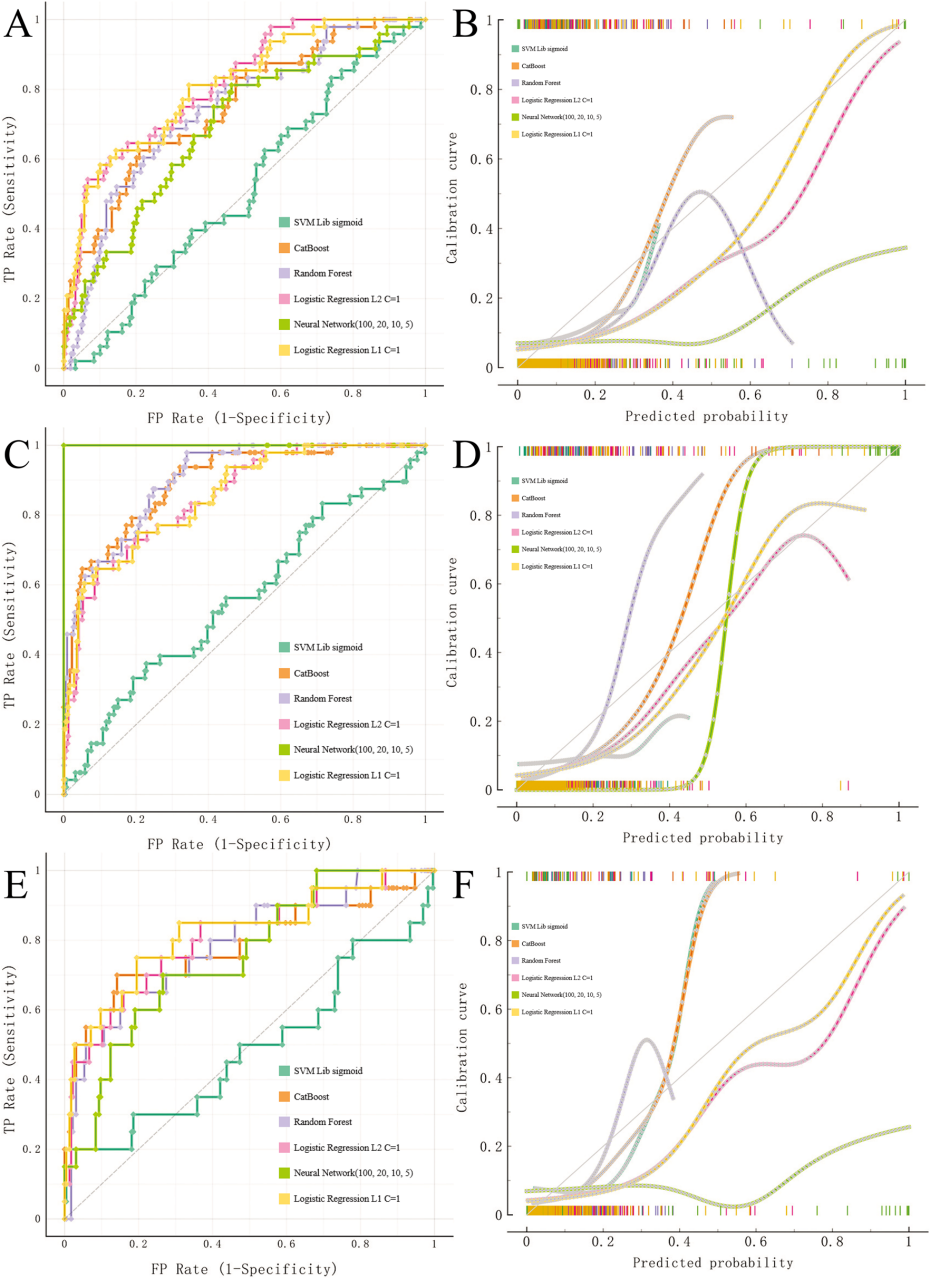
Model performance. Fivefold cross-validation was used to evaluate model performance in the training set. ROC curves and calibration curves were used to compare the strengths and weaknesses of the models. (**A**,**B**) ROC andibration curves of the five machine learning models on the training set using fivefold cross-validation. (**C**,**D**) internal validation on the training set. (**E**,**F**) ROC andibration curves of the five machine learning models in the test set. From Supplementary Table 4, we can find that the model obtained by Logistic Regression (L1 regularization) performs best with AUC = 0.819, F1 = 0.357 in the validation set.

Multiple machine learning algorithms were used to construct the classification models. Following the same approach, we constructed a classification model based on the core nine features. We validated the performance of the model on the training set using fivefold cross-validation (Fig. 2A,B; Supplementary Table 1). Additionally, we performed internal validation on the training set (Fig. 2C,D; Supplementary Table 2). We then tested these models in validation data (Fig. 2E,F; Supplementary Tables 3, 4). The results showed that the best model was obtained with LR L1(C = 1) at the average level (AUC = 0.819, CA = 0.927, F1 = 0.912, Precision = 0.912, Recall = 0.927; Fig. 2E, Supplementary Table 3). For the prediction of positive events, LR L1(C = 1) also achieved the best results (AUC = 0.819, CA = 0.927, F1 = 0.357, Precision = 0.625, Recall = 0.250; Fig. 2F, Supplementary Table 4). The previous analysis showed that the prognostic model constructed using the core nine features had the best predictive effect.

### Assessing the interpretability of model predictions

**Figure 3:**
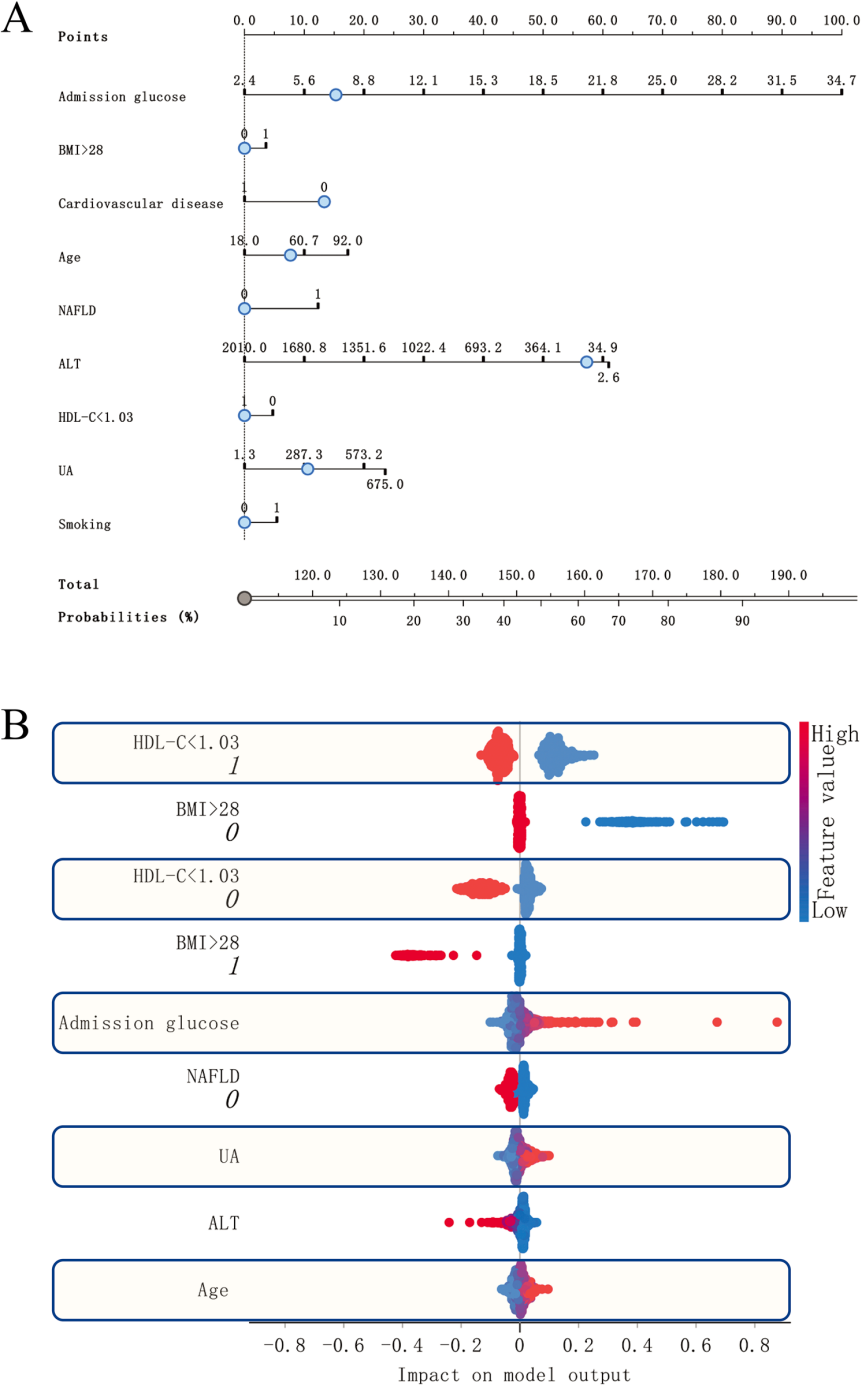
Model interpretation. We have used two methods of model interpretation. (**A**) Nomogram. The trend and magnitude of the effect of the nine core factors on the prediction of positive events can be observed in the figure. Admission glucose, BMI > 28, Age, NAFLD, UA and Smoking are the risk factors for PPDM-A. In contrast, Cardiovascular disease, ALT, HDL-C < 1.03 are negative predicted factors. (**B**) Sharpley value was used to explain the effect of the model on prediction. HDL-C < 1.03, BMI > 28 and Admission Glucose were the main factors affecting prediction. BMI > 28, Cardiovascular disease, HDL-C < 1.03 and Smoking are logistic variables, with 0 being FALSE and 1 being TRUE.

We constructed a nomogram based on LR L1(C = 1) for nine features. HDL-C < 1.03, CVD, and ALT were predicted to contribute to PPDM-A (Fig. 3A) negatively. To gain insight into the features that contributed most to the model prediction results, we used Sharply Values to assess the importance of core features for model evaluation (Fig. 3B). The factors that most impacted the model predictions included HDL-C < 1.03 mmol/l, BMI > 28 kg/m^2^, and Admission glucose. HDL-C < 1.03 = TRUE made the most significant contribution to predicting positive events. The contribution of BMI >28 = FALSE in predicting positive events was the opposite of BMI > 28 = TRUE. It suggests that obesity is also a causal factor for disease.

### Personalized diagnosis

**Figure 4:**
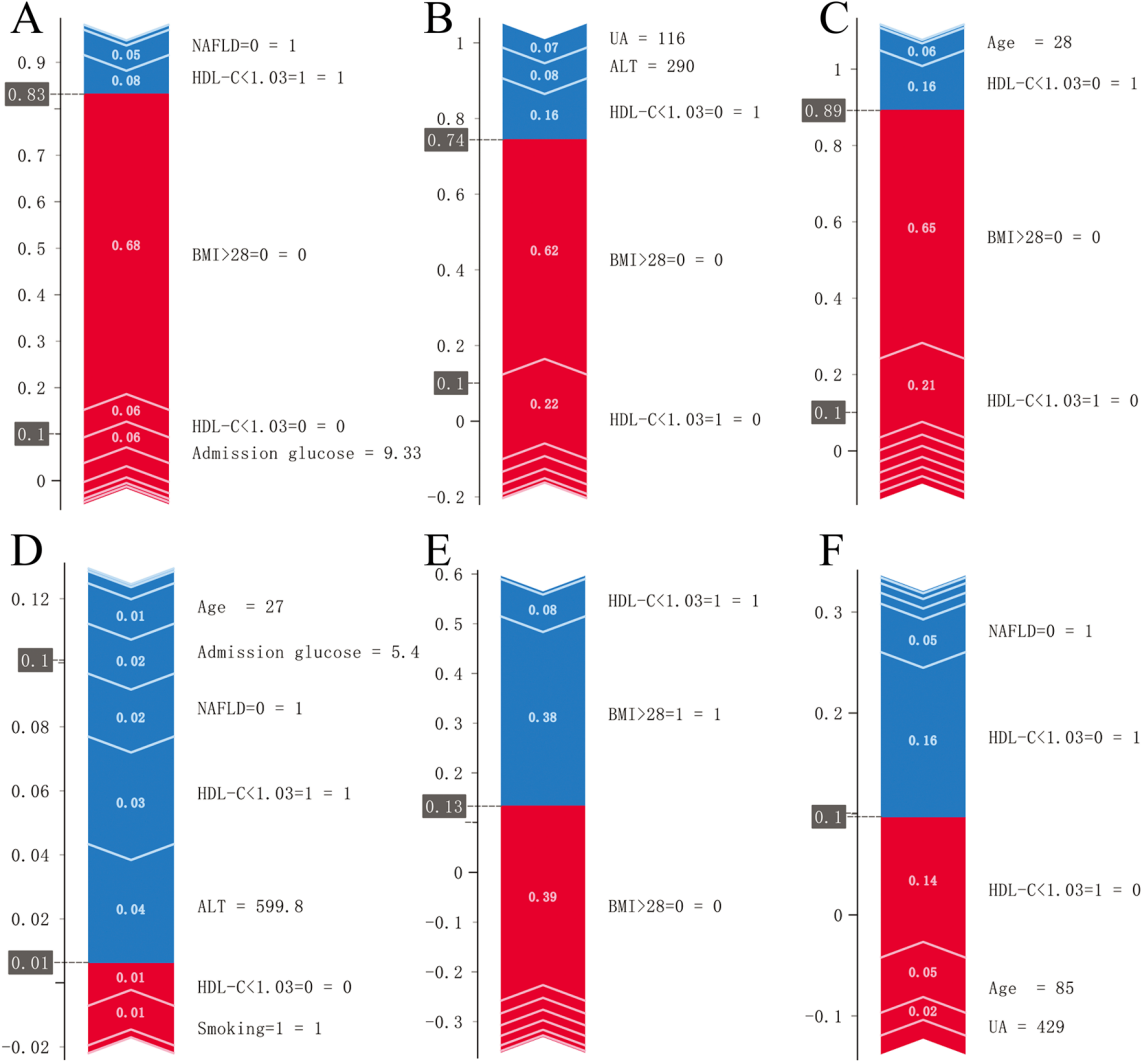
Personalized diagnosis. The risk factors for the three positive and three negative predicted samples in the prediction set were studied. (**A**) Patient 1 had a BMI > 28 kg/m^2^ as the main risk factor and a predicted probability of developing diabetes of 0.83. (**B**) Patient 2 had a BMI > 28 kg/m^2^, HDL-C > 1.03 mmol/l as the main factor and a predicted probability of developing diabetes of 0.74. (**C**) Patient 3 had a BMI > 28 kg/m^2^ as the main risk factor and a predicted probability of developing diabetes of 0.89. (**D**,**E**,**F**) Patient 4, Patient 5, Patient 6 have no factors that make a major contribution to predicting a positive event and all have a predicted probability of developing diabetes of less than 0.13. The contribution of risk factors to this patient can be observed in the graph. Red represents the positive contribution and blue represents the negative contribution.

We used the RL L1 (C = 1) model constructed with nine features to assess the main influences on the predictions of the six samples using Sharp Value. The results showed that the main contribution to an optimistic prediction for sample 1 came from (BMI > 28 = 0) = FALSE, (HDL-C < 1.03 = 0) = FALSE, with contributions of 0.68, 0.06 in order (Fig. 4A). The probability of an optimistic prediction for sample 1 was 0.83. The main contribution to an optimistic prediction for sample 2 came from (BMI > 28 = 0) = FALSE, (HDL-C < 1.03 = 1) = FALSE, with contributions of 0.62, 0.22 in order (Fig. 4B). The probability that this sample was predicted to be positive was 0.74. For sample 3, (BMI > 28 = 0) = FALSE, (HDL-C < 1.03 = 1) = FALSE contributed a positive predictive likelihood of 0.65, 0.21. Thus BMI > 28 kg/m^2^ was the leading risk factor for this sample (Fig. 4C). Multiple clinical information in samples 4, 5, and 6 contributed less to the positive prediction (Fig. 4D,E,F). The probability of predicting the occurrence of PPDM-A in each of these samples was less than 0.13.

## Discussion

PPDM-A is the most common sequela of pancreatitis^11,17,18^, and is characterized by poorer glycaemic control, a higher risk of developing cancer, and a higher risk of mortality^10,19–21^. However, the pathogenesis of diabetes secondary to acute pancreatitis is convoluted, which makes early clinical identification challenging. In addition, there is still no good classification model to predict PPDM-A in advance. Our feature contribution analysis prompted us to try to build a simpler predictive model based on a minimum number of the most influential features. To this end, we could fully evaluate the model’s performance using only nine pieces of information obtained about the patient. This study examined the ability to use nine clinical features to predict PPDM-A, leading to early intervention and effective PPDM-A screening.

Our results suggest that clinical features can accurately predict the risk of PPDM-A after the onset of acute pancreatitis, although none of the nine clinical features we included directly reflected islet cell function. The findings reveal that indicators related to pancreatic injury (APFC, PPC, ANC, WON, amylase) affected the predicted outcome during the feature selection process, consistent with previous studies^22,23^. However, growing evidence compels a reconsideration of the dogma: “β-cell destruction is the only underlying mechanism of diabetes after acute pancreatitis”. Our study reveals that age, BMI, metabolic status, and comorbidities play different roles in individuals and may lead to opposite outcomes. It may be due to the mutual influence of organs on each other in the case of imbalanced glucose metabolism^24–26^. We assessed the characteristics that had the most profound impact on the model's predictions by Shapley value. We found that admission glucose, obesity (BMI > 28 kg/m^2^), and HDL-C < 1.03 mmol /l were the three factors that had the most significant impact on the outcome, and this result is consistent with the results of feature extraction. Prior studies also have demonstrated that hyperglycemia during hospitalization of acute critical illness is associated with emergent diabetes and identifying patients for subsequent diabetes screening. In a Scottish retrospective cohort study, 2.3% of patients with an emergency admission to a hospital without previously known diabetes were newly diagnosed with diabetes within 3-years^27^. In a nationwide national cohort of consecutive patients with acute myocardial infarction without known diabetes, hyperglycemia at admission was significantly associated with subsequent diabetes (odds ratio: 2.56; 95% CI 2.15–3.06)^28^. Furthermore, changes in lipid metabolism and abnormal distribution of abdominal adipose tissue are significantly associated with PPDM-A.

Our work has several clinical applications. Firstly, it can facilitate early intervention in patients at high risk of diabetes. Early intervention for the development of diabetes is not currently studied. However, based on the health management knowledge of type 2 diabetes^29–32^, we can assume that a combination of diet and exercise can significantly decrease the incidence of diabetes. Due to the low prevalence of PPDM-A, early analysis of the effectiveness of prevention strategies can present some challenges. Our model can identify and recruit people at high risk of developing PPDM-A above 70%. Therefore, the current study paves the way for future randomized controlled trials to investigate further the effectiveness of using the model for early prediction of PPDM-A and possible prevention interventions. Another influential application is to help construct effective screening methods for PPDM-A. The prevalence of PPDM-A can already be confirmed considerably by admission glucose, BMI metrics, and HDL-C at the onset of acute pancreatitis. This staged risk assessment model can be used in subsequent studies to construct more rational design protocols for prospective studies of PPDM-A. Finally, by using the Sharpely Value, we can predict the likelihood of PPDM-A occurring in patients and identify key causative factors that can be targeted to give personalized treatment recommendations.

## Limitations

Our study has several limitations. Firstly, our model collected HIS data with inherent bias retrospectively from a small number of centers in urban China. Although we have tried to make use of existing knowledge about diabetes and AP in the selection of features in the HIS data, there are additional clinical features that may have been overlooked. These features may have better predictive effects. In addition, our overall data volume was inadequate. Although the sample size requirements for making inferences about the occurrence of PPDM-A using nine clinical information may be reduced, our model obtained low F1 and recall rates in predicting positive events, and these may have led to the under-recording of positive samples. Finally, the population to which the study applies is limited to HIS data information from the Chinese population, and the predictive value of its findings on other populations requires more data accumulation.

In conclusion, our work shows that it is possible to make accurate predictions of PPDM-A early in the onset of AP through nine clinical variables. These results may have many implications for the health of patients with PPDM-A. Our predictive model could form the basis for diagnosis and selective screening for PPDM-A and allow for personalized advice to patients on PPDM-A prevention. Future prospective studies, as well as multicentre, multicohort prospective studies, are needed to assess the clinical value of the model.

## Methods

### Study design and participants

This multicentre cross-sectional follow-up study included all consecutive patients with first-episode AP admitted to Zhongda Hospital of Southeast University, Yixing Second People’s Hospital, First Affiliated Hospital of Xinjiang Medical University, and Hunan Provincial People’s Hospital from 1 October 2016 to 31 October 2021. The study was approved by the ethics committee of Zhongda Hospital, affiliated with Southeast University, and performed according to the Declaration of Helsinki and relevant regulations. Informed consent was obtained orally from all participants.

Inclusion criteria were as follows:

1. Diagnosis of AP based on international guidelines^33^;
2. Age ≥ 18 years;
3. Admitted with abdominal pain for < 48 h.

Exclusion criteria were as follows:

1. Recurrent AP;
2. Chronic pancreatitis;
3. History of diabetes, HbA1c ≥ 6.5% or hypoglycemic drugs diagnosed before AP attack;
4. History of malignant tumor;
5. Severe heart, liver, kidney and other organ dysfunction;
6. History of immune system diseases or hormone use;
7. Mental illness, unable to cooperate with research;
8. Pregnancy and lactation;
9. Data missing > 10%
10. Death during admission.

### Data collection

Date of demographic parameters (gender, age), Family history of diabetes, smoking, drinking, Clinical comorbidities (CVD, NAFLD, and hypertension), vital signs (systolic blood pressure, diastolic blood pressure, body mass index (BMI)), laboratory studies (amylase, admission glucose, serum calcium, hepatic and renal functions, lipid profiles), severity and etiology of AP, length of stay, infection condition, local complications and systemic complications of AP were extracted through HIS.

### Definitions and classification

BMI was calculated as weight (kg) divided by the square of height (m). According to the Guidelines for Prevention and Control of Overweight and Obesity in Chinese adults, obesity was defined as BMI greater than or equal to 28 kg/m^2^. The severity of AP was defined as mild, moderately severe, and severe according to the revised Atlanta classification^33^. Local complications include acute peripancreatic fluid collection(APFC), pancreatic pseudocyst(PPC), acute necrotic collection(ANC), and walled-off necrosis (WON)^33^. Signs of systemic inflammatory response syndrome (SIRS) defined by presence of two or more criteria: 1. Heart rate > 90 beats/min; 2. Core temperature < 36 °C or > 38 °C; 3. White blood count < 4000 or > 12,000/mm^3^; 4. Respirations > 20/min or PCO2 < 32 mm Hg. PPDM-A was defined as new onset diabetes more than 90 days after AP with no history of diabetes before the AP episode^14,34^ and absence of type 1 diabetes–associated autoimmunity. Organ failure is defined as a score of 2 or more for one of these three organ systems using the modified Marshall scoring system^35^.

### Statistical analysis

Logistic regression L1 regularization was used to screen for appropriate features. We extracted features with weights > 0.01 as core classification features. The top 9 features were obtained for the follow-up study.

We constructed five common machine learning algorithms using Orange3^36^: logistic regression, neural networks, random forests, catBoost^37^, and SVM. Fivefold cross-validation was used to evaluate the predictive power of the model. Five metrics, Area under ROC(AUC), Classification accuracy (CA), F1, accuracy, and recall, were used to evaluate the model. AUC is the area under the receiver-operating curve. The larger the AUC, the better the model effect. CA is the proportion of correctly classified samples. F1 is a weighted harmonic mean of precision and recall. F1 can be used to evaluate the model's trade-off between precision and recall metrics. Precision is the proportion of true positives among instances classified as positive. Recall is the proportion of true positives among all positive instances in the data. In assessing the model's effectiveness, we primarily used ROC and Calibration curves in the test set to assess the predictive effectiveness of the model. We analyzed the average performance of the model ground and the performance of the predicted positive events separately.

To understand the relationship between individual features and model output, we use Shapley values^38^, which can be used to evaluate the outcome of complex models and are particularly applicable to artificial neural networks and gradient boosting machines (CatBoost). By averaging over all samples, the Shapley values estimate the contribution of each feature to the overall model prediction. In addition, Shapley analysis allows the contribution of each individual risk factor to the diagnosis to be assessed.

## Supplementary Information

Supplementary Information.

## Data Availability

All the data are available upon request to the corresponding author.
