# Supplementary material for: Machine learning for post-acute pancreatitis diabetes mellitus prediction and personalized treatment recommendations
Source: Sci Rep. 2023 Mar 24;13:4857. doi: 10.1038/s41598-023-31947-4 (PMC10038980; doi:10.1038/s41598-023-31947-4)
Supplement: Supplementary file 1 — Supplementary Information. [file 41598_2023_31947_MOESM1_ESM.docx]

Supplementary Fig1. Data Cleaning Process


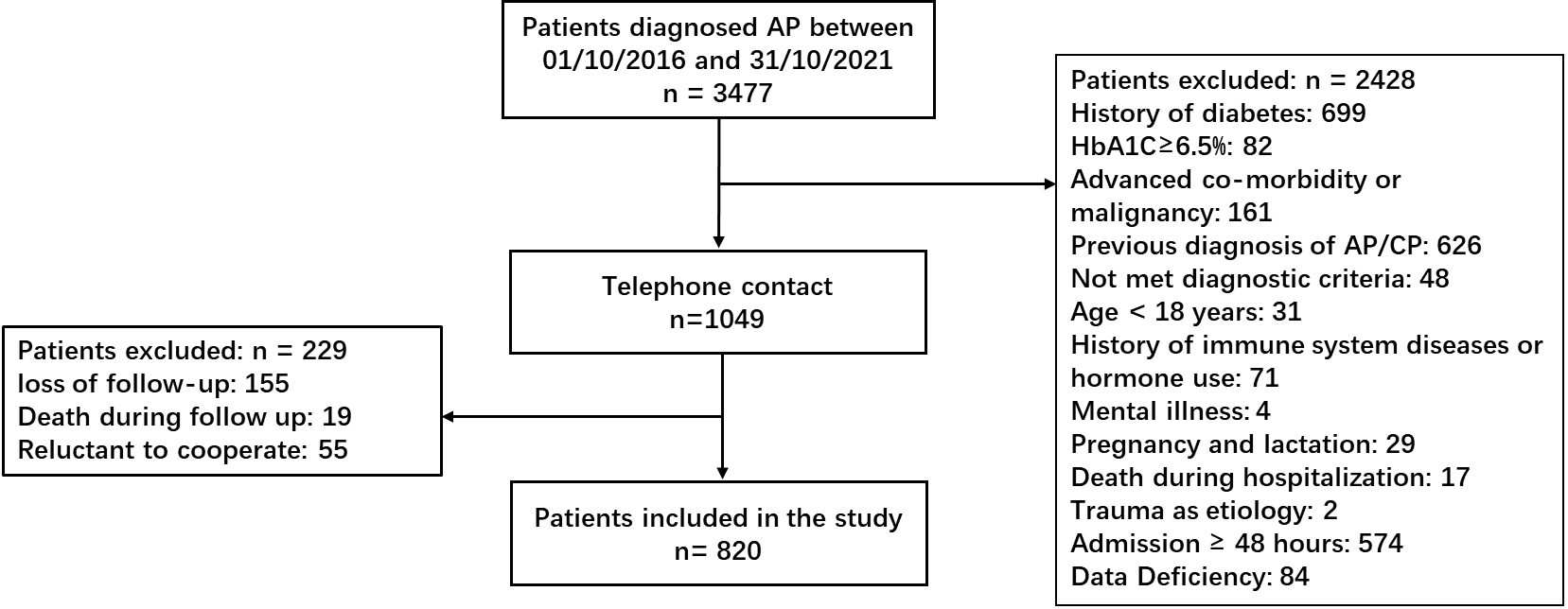


Supplementary Table 1. Training data 5-fold cross-validation average performance

| **Model** | **AUC** | **CA** | **F1** | **Precision** | **Recall** |
| --- | --- | --- | --- | --- | --- |
| LR L1 C=1 | 0.811 | 0.927 | 0.904 | 0.918 | 0.927 |
| LR L2 C=1 | 0.813 | 0.915 | 0.887 | 0.882 | 0.915 |
| Random Forest | 0.740 | 0.913 | 0.875 | 0.839 | 0.913 |
| Gradient Boosting | 0.752 | 0.923 | 0.892 | 0.929 | 0.923 |
| Neural Network (100,20,10,5) | 0.693 | 0.880 | 0.875 | 0.871 | 0.880 |
| SVM | 0.519 | 0.869 | 0.860 | 0.851 | 0.869 |

Supplementary Table 2. Average performance in Training data

| **Model** | **AUC** | **CA** | **F1** | **Precision** | **Recall** |
| --- | --- | --- | --- | --- | --- |
| LR L1 C=1 | 0.854 | 0.929 | 0.905 | 0.926 | 0.929 |
| LR L2 C=1 | 0.851 | 0.922 | 0.894 | 0.907 | 0.922 |
| Random Forest | 0.902 | 0.916 | 0.876 | 0.840 | 0.916 |
| Gradient Boosting | 0.894 | 0.927 | 0.900 | 0.932 | 0.927 |
| Neural Network (100,20,10,5) | 1.000 | 1.000 | 1.000 | 1.000 | 1.000 |
| SVM | 0.560 | 0.864 | 0.857 | 0.850 | 0.864 |

Supplementary Table 3. Average performance in validation data

| **Model** | **AUC** | **CA** | **F1** | **Precision** | **Recall** |
| --- | --- | --- | --- | --- | --- |
| LR L1 C=1 | 0.819 | 0.927 | 0.912 | 0.912 | 0.927 |
| LR L2 C=1 | 0.805 | 0.915 | 0.895 | 0.888 | 0.915 |
| Random Forest | 0.788 | 0.919 | 0.880 | 0.844 | 0.919 |
| Gradient Boosting | 0.783 | 0.927 | 0.898 | 0.932 | 0.927 |
| Neural Network (100,20,10,5) | 0.751 | 0.878 | 0.875 | 0.872 | 0.878 |
| SVM | 0.489 | 0.862 | 0.865 | 0.868 | 0.862 |

Supplementary Table 4. Positive predictive performance in validation data

| **Model** | **AUC** | **CA** | **F1** | **Precision** | **Recall** |
| --- | --- | --- | --- | --- | --- |
| LR L1 C=1 | 0.819 | 0.927 | 0.357 | 0.625 | 0.250 |
| LR L2 C=1 | 0.805 | 0.915 | 0.222 | 0.429 | 0.150 |
| Random Forest | 0.788 | 0.919 |  |  |  |
| Gradient Boosting | 0.783 | 0.927 | 0.182 | 1.000 | 0.100 |
| Neural Network (100,20,10,5) | 0.751 | 0.878 | 0.211 | 0.222 | 0.200 |
| SVM | 0.489 | 0.862 | 0.190 | 0.182 | 0.200 |
